# The relationship between dietary intake and stunting among pre-school children in Upper Egypt

**DOI:** 10.1017/S136898002100389X

**Published:** 2022-08

**Authors:** Eman Mohamed Mahfouz, Eman Sameh Mohammed, Shaza Fadel Alkilany, Tarek Ahmed Abdel Rahman

**Affiliations:** Public Health and Preventive Medicine Department, Faculty of Medicine, Minia University, Minia 61519, Egypt

**Keywords:** Stunting, Pre-school children, Dietary intake, Egypt

## Abstract

**Objective:**

Linear growth is controlled by several factors, malnutrition is one of the leading causes of stunted child growth. The objective of this study was to determine the dietary intakes associated with stunting among pre-school children in rural Upper Egypt.

**Design:**

Community-based cross-sectional study

**Setting:**

Data were collected by interviewing the children’s caregivers in the rural household setting.

**Participants:**

The study included 497 pre-school children aged 2–5 years in rural Upper Egypt. Food intake data were estimated using 24-h recall method. Anthropometric measurements of children were taken and then converted to z-scores for weight-for-age Z-score, height-for-age Z-score and weight-for-height Z-score.

**Results:**

The study included 497 children of which 19·1 % were stunted, 76·3 % did not meet recommended energetic intake and 13·7 % did not meet recommended protein intake and this was significantly higher than non-stunted children. Children who were stunted significantly consumed poultry, eggs and fruits less often than non-stunted children, by regression; male sex (adjusted odds ratio (aOR) = 1·91), mother’s age (0·93), lower socio-economic status (SES); and not meeting recommended protein intake (aOR = 2·26) were found to be associated with stunting.

**Conclusion:**

Male sex, younger mothers, lower SES and not meeting recommended energy and protein were statistically associated with stunting. Nutrition education messages encouraging adequate and healthy eating are recommended.

Growth was previously identified as a ‘mirror of the conditions of society’, especially the ‘nutritional and hygienic status’ of the population^(1)^. Stunting (short stature for age) is the most commonly used indicator of chronic malnutrition and is assessed by anthropometric measures of the child’s height for age^(2)^. Globally, the prevalence of stunting fell from 29·5 %t to 22·9 % between 2005 and 2016, although 155 million children under 5 years of age across the world still suffer from stunted growth, ranging from nearly 2 % in high-income countries to more than 50 % in low-income countries^(3,4)^. According to Egypt Demographic and Health Survey (EDHS) 2014, one in five Egyptian children under 5 years of age was stunted (short for their age) and 24·8 % of children of rural Upper Egypt were stunted^(5)^. In Minia, it was found that 20·3 % of children aged 6–24 months were stunting^(6)^.

Linear growth is controlled by complex genetic, physiological and nutrient-sensitive endocrine/paracrine/autocrine-mediated molecular signalling mechanisms, possibly including sleep adequacy through its influence on growth hormone secretion^(7)^.

In global settings of poverty, malnutrition is often driven by energetic scarcity which, along with infection, is one of the leading causes of stunted child growth^(8)^. Deficiency in protein and Zn is associated with poverty and results in decreased linear growth. Zn is found in red meat and poultry and humans do not have Zn tissue reserves. Therefore, when dietary intake is inadequate, a child’s linear growth decreases^(9)^. Other micronutrients deficiency like vitamin A and Fe deficiencies cause growth flattering when the deficiency is severe^(10)^.

Childhood stunting is associated with increased risk for adult diseases (e.g. obesity, CVD and diabetes), and with suboptimal brain development, which leads to impaired cognitive ability and school performance, and reduces earning potential later in life^(9)^.

One study demonstrated that high consumption of animal products was associated with the decreased risk of maternal–child double burden which was defined by the coexistence of maternal overweight and child stunting within the same household. Therefore, improving child stunting through adequate intake of animal products is critical to solve the problem of maternal–child double burden^(11)^.

Egypt has floated its currency in November 2016, leading to reduction of its value by almost 50 % against the dollar. Although the liberalisation should help the country to strengthen its economy, it makes life harder for Egyptians because the cost of goods and price of foods has risen sharply over the past few years^(12)^. Assessing relationship between stunting and dietary pattern is important, especially after floating of Egyptian currency. Early deficits in childhood growth and development contribute to long-term problems that can persist into adulthood^(13)^. Understanding of the patterns and factors associated with stunting could enable nutrition programmes to target nutritionally vulnerable individuals.

The objective of this study was to determine dietary intakes associated with stunting among pre-school children in rural Upper Egypt.

## Methods

### Study design and participants

This cross-sectional study was conducted during the period from November 2017 to March 2018. The study population consisted of children aged 2–5 years of age and their caregivers in rural Upper Egypt. Children having chronic diseases (e.g. cerebral palsy, asthma or diabetes, cardiac, renal or liver diseases) or taking medications that may affect their dietary intake and/or the overall nutritional status were excluded. In households with more than one child aged 2–5 years, the youngest child was selected.

### Sampling methods and sample size

A random sampling was done to select one district out of the nine districts of the studied area; then one village was chosen randomly, considering that the district had homogenous characteristics. All households with a child aged 2 to 5 years of age had an equal chance of being asked to participate in the survey.

A required sample size of 258 children was estimated taking into account prevalence of stunting in Egypt of 21·4 % based on Egypt demographic health survey^(14)^ to provide 96 % power at the level of 5 % significance using the statistical software EPI-INFO 7.2.2.6. The actual sample collected was 497 children. Non-response rate was 4·1 %

### Data collection

Data were collected by face-to-face interviews in the household setting using a multi-component questionnaire; the aim of the study was explained. With the help of the local health facilities of the chosen areas, two health visitors contacted the target families. At the visit, the investigators introduced themselves to the head of the household and obtained verbal approval for participation in the study.

### Measures

The items in the questionnaire included:

### Anthropometric measures

Digital scale was used to measure weight to the nearest 0·1 kg. A stretch-resistant measuring tape was used to measure standing height to the nearest 0·5 cm. A big, flat, set square was used to make a right angle with the wall to ensure that height was measured accurately. Participants were asked to remove shoes and bulky clothes before measurements^(15)^. Mid-upper arm circumference (MUAC) was measured on the right arm using a non-elastic tape held midway between the acromion and the olecranon processes, with arm hanging loosely at the side of the body. MUAC-for-age *z*-score (MUACZ) was calculated using *WHO Anthro* software (version 3·2·2.)^(16)^.

#### Conversion of weight and height to Z-score

Each child’s height-for-age Z-score (HAZ), weight-for-age Z-score, weight-for-height Z-score, BMI-for-age Z-score and MUACZ for age and sex were calculated based on WHO Child Growth Standards software WHO Anthro (version 3.2.2, January 2011)^(16)^. Children were classified as being stunted, underweight or wasted on the basis of their HAZ, weight-for-age Z-score and weight-for-height Z-score, respectively, when their score was 2 sd below the reference median according to the WHO^(17)^. The mean HAZ/weight-for-age Z-score/weight-for-height Z-score/BMI-for-age Z-score/MUACZ was calculated as well as the proportion of children 2 sd below the reference level.

### Dietary intake

Data on diet had been collected using specially designed questionnaires to cover required information on: food intake (24-h recall) and dietary pattern ‘food frequency’ for selected items.

### 24-h dietary recall

Food consumption was assessed by quantitative 24-h dietary recall method applied on the past 24 h during a personal interview. In this method, mothers were asked to recall the exact foods and beverages her child consumed during the previous 24-h period, from the first intake in the morning until the last foods or beverages consumed at night (before going to bed or later, in the case of those who get up at midnight and eat and/or drink something)^(18)^. Quantities of food and beverages were estimated using cups and household utensils commonly used then converted into grams^(19)^.

Nutrient analysis and the calculation of diet energy intake were performed using the software program *NutriSurvey*^(20)^, mixed-dishes not found in *NutriSurvey* databases were deconstructed into its basic constituents. Dietary components assessed included energy intake as kilojoules, intake of total fat grams per day (g/d), total carbohydrates (g/d) and proteins (g/d). Energy deficit for children was calculated by taking the difference between children intake of total energy and recommended daily allowance^(21)^.

### Food intake frequency

Data were collected on the usual intake of commonly consumed foods during the prior 12 months (during the year followed floating of the Egyptian currency). The food items included meat, poultry, fish, egg, milk, milk products, fruits, vegetables, legumes, rice/macaroni and tubers. Selected food items were chosen based on commonly consumed foods validated using public health expert’s knowledge about culturally specific foods. Intakes of different food items were assessed using short answer questions that asked ‘How often do you eat each item per week?’ Food frequency categories ranged from never or less than once per month to every day^(22)^.

### Socio-economic status

Socio-economic status (SES) was calculated according to El-Gilany *et al.* (2012)^(23)^, a modification of the old scoring system of Fahmy and El-Sherbini (1983)^(24)^. The scale has seven domains with a total score of 84, with a higher score indicating better SES. Total score was calculated by summing the score of the seven domains: education and cultural, occupation, family, family possessions, economic, home sanitation, and health care.

SES was classified to very low (score < 35), low (score 35–41), middle (score 42–47) and high (score ≥ 48) depending on the quartiles of the calculated score rather than a fixed point.

### Other measures

The questionnaire included questions about mother’s age, child’s sex, number of children in the house and child birth order. Perceived size at birth was reported by mother, they were asked to put it in one category (lower than average, normal/average and higher than average).

### Statistical analysis

Participants with missing information for key variables were excluded from the analysis. Normality of the data was tested using the Kolmogorov–Smirnov tests. Data are presented as mean and standard deviation. Comparison between unrelated variables was conducted with Student’s *t* test. The chi-square and Fisher’s exact tests were used for comparison between categorical variables. Binary logistic regression analysis was used to determine which factors were significantly and independently associated with stunting after adjustment for potential confounders. Factors found to be significantly associated with stunting by univariate analysis were entered into the multivariable model. Several variables were not entered into the model due to multicollinearity and highly correlated with SES as they are part of socio-economic score (income, education and occupation of parents)

Significance was accepted at *P* < 0·05. Statistical analyses were conducted using the IBM Statistical Package for the Social Sciences (IBM SPSS v.20; IBM Corporation Inc).

## Results

The study included 497 children aged 2–5 years who had their height and weight measured. Among the studied children, 95 (19·1%) were stunted. Table 1 outlines the demographic characteristics of the study population and compares between stunted and non-stunted children. Children respondents’ age ranged between 24 and 60 months with a mean of 40·1 months (sd = 11·1), and the ratio of males to females was almost fifty-fifty. The perceived size at birth was lower than average for 14·3% of the studied children. Approximately one-fourth of the studied children was first-born child and one-fourth was second-born, while half of the children were ordered the third child or more. About 24 % children were born before their elder sibling completed 2 years. Stunting was higher among male children as compared to female children. University graduates and postgraduates accounted for 9·2% of mothers and 17·7% of fathers of non-stunted children which were higher than 1·1% and 5·3%, respectively, in children who were stunted (*P* < 0·001).

**Table 1:** Demographic characteristics of the studied children

	Total (*n* 497)			Stunted (*n* 95)			Not stunted (*n* 402)			*P*-value
	*n*	%	Range	*n*	%	Range	*n*	%	Range	
Age (months)										
Mean	40·1		24·02–59·99	39·22		24·02–59·99	40·34		24·02–59·99	0·378
sd	11·1			10·08			11·35			
Sex										
Male	248	49·9		58	61·1		190	47·3		0·016
Female	249	50·1		37	38·9		212	52·7		
Perceived size at birth										
Lower than average	71	14·3		9	9·5		62	15·4		
Normal/average	416	83·7		84	88·4		332	82·6		0·329
Higher than average	10	2·0		2	2·1		8	2·0		
Child birth order										
1st	129	26·0		28	29·5		101	25·1		
2nd	119	23·9		28	29·5		91	22·6		0·166
3rd	126	25·4		23	24·2		103	25·6		
4th or more	123	24·7		16	16·8		107	26·6		
Mother’s education										
Illiterate	117	23·5		23	24·2		94	23·4		
Below 2ry	82	16·5		22	23·2		60	14·9		0·020
2ry, institute	260	52·3		49	51·6		211	52·5		
University, post	38	7·6		1	1·1		37	9·2		
Father’s education										
Illiterate	77	15·5		16	16·8		61	15·2		
Below 2ry	91	18·3		33	34·7		58	14·4		< 0·001
2ry, institute	265	53·3		41	43·2		224	55·7		
University, post	64	12·9		5	5·3		59	14·7		
Mother’s work										
Housewives	445	89·5		89	93·7		356	88·6		0·142
Working	52	10·5		6	6·3		46	11·4		
Occupation of father										
Non-working	7	1·4		0	0·0		7	1·7		
Unskilled	97	19·5		22	23·2		75	18·7		0·253
Skilled	266	53·5		56	58·9		210	52·2		
Business	48	9·7		8	8·4		40	10·0		
Semiprofessional	45	9·1		6	6·3		39	9·7		
Professional	34	6·8		3	3·2		31	7·7		
Income										
In debt	54	10·9		17	17·9		37	9·2		
Routine	85	17·1		29	30·5		56	13·9		< 0·001
Routine + emergency	184	37·0		31	32·6		153	38·1		
Save money	174	35·0		18	18·9		156	38·8		
Socio-economic level										
Very low (< 35)	119	23·9		32	33·7		87	21·6		
Low (35–41)	108	21·7		30	31·6		78	19·4		< 0·001
Middle (42–47)	136	27·4		19	20·0		117	29·1		
High (≥ 48)	134	27·0		14	14·7		120	29·9		

Table 2 shows that a total of 19·1 % of the studied children were stunted (HAZ < –2), 1·6 % were wasted and 1·8 % were underweight. Overweight or obese children (weight-for-height Z-score > +2) accounted for 9·5 %. Nearly, 14 % were obese (BMI-for-age Z-score > +2).

**Table 2:** Anthropometric measurements of the participating children

	Mean	sd	Range	< –2 sd		+2 to –2 sd		> +2 sd	
				*n*	%	*n*	%	*n*	%
HAZ	−1·03	1·15	4·97 to 3·65	95 (stunted)	19·1	399	80·3	3	0·6
WHZ	0·62	1·06	−3·34 to 3·96	8 (wasted)	1·6	442	88·9	47	9·5
WAZ	−0·15	0·86	−3·39 to 2·24	9 (underweight)	1·8	486	97·8	2	0·4
MUACZ	0·2	0·87	−2·89 to 3·09	10	2	477	96	10	2
BAZ	0·74	1·1	−3·54 to 4·44	9	1·8	418	84·1	70	14·1

WAZ, weight-for-age Z-score; HAZ, height-for-age Z-score; WHZ, weight-for-height Z-score; MUAC, mid-upper arm circumference; BAZ, BMI-for-age Z-score.

As shown in Table 3, stunted children of 2–3 years age group had lower mean daily intake of energy compared to non-stunted children (4121·3 ± 949 kJ compared to 4516·1 ± 1077·1 kJ, respectively) and this difference was statistically significant. In age group of 4–5 years, the mean daily intake of protein and carbohydrates were lower in children who were stunted compared to non-stunted.

**Table 3:** Dietary intake of children using 24-h recall

Children (2–3 years)	Total (*n* 204)			Stunted (*n* 43)			Not stunted (*n* 161)			*P*-value
	Mean	sd	Range	Mean	sd	Range	Mean	sd	Range	
Energy (kJ/d)	4432·9	1103·6	2092·9–6939·3	4121·3	949	2439·1–6939·3	4516·1	1077·1	2093·1–6902·3	0·037
Protein (g/d)	28·18	9·9	10·02–60·68	26·21	10·84	10·03–47·79	28·7	9·6	10·02–60·68	0·143
Fat (g/d)	36·75	11·86	6·66–70·83	34·99	12·02	6·66–65·9	37·21	11·82	10·25–70·83	0·276
Carbohydrates (g/d)	151·04	48·31	58·66–310·19	140·49	45·07	73·15–226·99	153·86	48·88	58·66–310·19	0·107

	Total (*n* 293)			Stunted (*n* 52)			Not stunted (*n* 241)			
Children (4–5 years)	Mean	sd	Range	Mean	sd	Range	Mean	sd	Range	*P*-value
Energy (kJ/d)	4891·4	1099·1	2261–7735·4	4389·7	1054·6	2260·8–7108·2	4999·7	1080·3	2348·5–7735·4	< 0·001
Protein (g/d)	32·41	9·84	4·2–67·55	29·1	9·04	12·35–47·45	33·12	9·88	4·2–67·55	0·007
Fat (g/d)	40·14	12·33	10·25–78·73	37·78	10·94	16·83–63·34	40·66	12·57	10·25–78·73	0·127
Carbohydrates (g/d)	170·08	47·72	58·66–314·88	147·11	43·13	69·32–273·16	175·04	47·29	58·66–314·88	< 0·001

	Total (*n* 497)			Stunted (*n* 95)			Not stunted (*n* 402)			
	Mean	sd		Mean	sd		Mean	sd		*P*-value
Not meeting recommended energetic intake*	279	56·1 %		66	76·3 %		213	23·7 %		0·004
Not meeting recommended protein intake*	37	7·4 %		13	13·7 %		24	6 %		0·01

DRI, dietary reference intake.

* Based on DRI^(25)^.

DRI for children aged 2–3 years: 4180 kJ (1000 kcal) and 13 g protein.

DRI for children aged 4–5 years: 5016 kJ (1200 kcal) and 19 g protein.

Approximately 56 % of the children did not achieve the energy recommendations, while 7·4 % did not achieve the protein recommendations. Among stunted group, significantly more children did not meet dietary reference intake for energy (76·3 %) and protein (13·7 %) compared to non-stunted group (23·7 % and 6 %, respectively).

Table 4 shows that children who were stunted significantly consumed poultry (*P* = 0·001), eggs (*P* = 0·027) and fruits (*P* = 0·001) less often than non-stunted children, while consumed legumes more frequently (*P* = 0·014). There were no associations between other food groups and stunting.

**Table 4:** Average weekly food consumption frequency: compared by stunting in children

Weekly food group intake frequency (times/week)	Total (*n* 497)			Stunted (*n* 95)			Not stunted (*n* 402)			OR	95 % CI	*P*-value
	Mean	sd	Range	Mean	sd	Range	Mean	sd	Range			
Meat	0·57	0·71	0–3·5	0·47	0·68	0–3·5	0·59	0·72	0–3·5	0·77	0·54, 1·09	0·133
Poultry	1·53	0·82	0–5·5	1·26	0·77	0–3·5	1·59	0·82	0–5·5	0·56	0·41, 0·77	< 0·001*
Fish	0·19	0·43	0–2	0·23	0·47	0–2	0·18	0·42	0–2	1·26	0·78, 2·03	0·34
Egg	3·12	2·09	0–7	2·69	2	0–7	3·22	2·1	0–7	0·88	0·79, 0·99	0·028*
Milk	1·53	2·24	0–7	1·44	2·42	0–7	1·55	2·19	0–7	0·98	0·88, 1·08	0·671
Milk products	3·61	2·22	0–7	3·56	2·41	0–7	3·62	2·17	0–7	0·99	0·89, 1·09	0·822
Fruits	2·81	2·10	0–7	2·08	1·69	0–7	2·98	2·15	0–7	0·78	0·69, 0·89	< 0·001*
Vegetables	3·98	2·00	0·63–7	3·84	2·09	0·63–7	4·02	1·98	0·63–7	0·96	0·85, 1·07	0·436
Legumes	5·51	2·04	0·25–7	5·97	1·78	1–7	5·4	2·09	0·25–7	1·17	1·03, 1·32	0·016*
Rice/macaroni	3·73	1·63	0·25–7	4·01	1·96	0·25–7	3·67	1·54	0·25–7	1·14	0·99, 1·3	0·061
Tubers (potatoes)	4·67	1·67	0–7	4·92	1·66	1–7	4·61	1·67	0–7	1·12	0·98,1·29	0·107

* Significant difference at *P*-value < 0·05.

Table 5 reveals that sex, mother’s age, SES and not meeting recommended energy and protein were significantly associated with stunting. Male children were more likely to be stunted than female children (adjusted odds ratio (aOR) = 1·91, 95 % CI = 1·17, 3·1). The increase in age of the mother by 1 year was associated with 7 % decrees in the odds of child to be stunted (aOR = 0·93, 95 % CI = 0·88, 0·99). Children from very low and low socio-economic households were more likely to be stunted compared to children from high socio-economic households (aOR = 3·05, CI = 1·45, 6·39 and aOR = 2·74, CI = 1·31, 5·72, respectively). Low protein intake was associated with stunting (aOR = 2·26, CI = 1·01, 5·05).

**Table 5:** Binary logistic analysis of factors associated with stunting among the studied sample

	Stunting					
	Crude OR	95 % CI	*P*-value	Adjusted OR	95 % CI	*P*-value
Child sex						
Female	1·00 (reference)			1·00 (reference)		
Male	1·75	1·11, 2·76	0·016*	1·91	1·17, 3·10	0·009*
Height of mother	0·94	0·9, 0·98	0·002*	0·97	0·92, 1·01	0·139
Age of mother	0·93	0·89, 0·97	0·001*	0·93	0·88, 0·99	0·018*
SES						
Very low	3·15	1·59, 6·26	0·001*	3·05	1·45, 6·39	0·003*
Low	3·3	1·65, 6·61	0·001*	2·74	1·31, 5·72	0·007*
Middle	1·39	0·67, 2·9	0·378	1·27	0·59, 2·73	0·537
High	1·00 (reference)			1·00 (reference)		
Not meeting recommended protein intake	2·5	1·22, 5·11	0·012*	2·26	1·01, 5·05	0·047*
Not meeting recommended energetic intake	2·02	1·25, 3·26	0·004*	1·65	0·98, 2·77	0·06
No. of children	0·81	0·68, 0·98	0·029*	0·95	0·75, 1·21	0·684
Perceived size at birth						
Lower than average	0·58	0·11, 3·18	0·531			
Normal/average	1·01	0·21, 4·85	0·988			
Higher than average	1·00 (reference)					
Child birth order						
1st	1·00 (reference)					
2nd	1·11	0·61, 2·01	0·731			
3rd	0·81	0·44, 1·49	0·491			
4th or more	0·54	0·28, 1·06	0·072			

N.B. Dependent variable stunting, SES socio-economic status.

*R*^2^ = 0·158.

* Significant difference at *P*-value < 0·05.

## Discussion

Nutritional status is a primary determinant of a child’s health and well-being. The prevalence of stunting among under 5 years of age children in the current study was 19·1 % with mean HAZ of –1·03. This was consistent with 2014 EDHS which reported that 21 % of children under 5 years of age were stunted with mean HAZ of –0·6^(14)^. Another similar finding was reported in Minia, where 20·3 % of children (age 6–24 months) were stunted^(26)^.

From the 95 children who were stunted, stunting was higher among male children as compared to female children (61·1 and 38·9 %, respectively). A similar finding was reported in 2014 EDHS^(14)^, where 22·8 % of males and 19·9 % of females were stunted, also^(26–28)^ reported a similar finding. Furthermore, a meta-analysis of Demographic and Health Surveys (DHS) data from 10 sub-Saharan Africa found that stunting prevalence was 46 % among boys, compared to 36 % among girls^(29)^. On the other hand, Mahmudiono et al. (2018) reported that female children under 5 years of age were less likely to be stunted than their male counterparts (OR: 0·612)^(30)^. The higher prevalence of stunting among males than females was also reported in Zambia (42·4 and 37·6 %, respectively)^(31)^.

One possible explanation is that nutritional requirements may increase in male as they are actively playing outside house more than females^(32)^. Moreover, higher incidence of rates infectious diseases common among infants and young children were reported to be higher in male children and was attributed to greater male mobility^(33)^.

Obese children (BMI-for-age Z-score > 2 sd) were 14·1 % similar to EDHS 2014 (14·9 %)^(14)^. The coexistence of undernutrition and overnutrition is referred to as the double burden of malnutrition (DBMN). Although apparently paradoxical, both can emerge from the same root causes: poverty and food insecurity^(34)^.

Regarding the studied children, in order to interpret the dietary data in the current study, children were grouped according to their age and dietary requirements into two groups, 2–3 years and 4–5 years of age.

In the present study, the mean energy intake for children aged 2–3 years was 4432·9 kJ, higher than 3569·7 kJ (854 kcal) that was reported in Kenya for the same age group. However, children aged 4–5 years consumed less energy intake (4891·4 kJ) than what was reported by the same study among children of the same age group (5793·5 kJ)^(35)^.

The study demonstrated that the mean daily intake of energy of both age groups were lower among children who were stunted compared to non-stunted children, while the mean daily intake of protein and carbohydrates were lower in stunted children of age group 4–5 years. A previous study among pre-school children of an urban slum community in Dhaka, Bangladesh, showed that the average daily dietary intake of energy, protein, carbohydrate and lipid were lower in stunted children compared to non-stunted children^(36)^.

Among stunted group significantly more children did not meet dietary reference intake for protein (13·7 %) compared to non-stunted group (6 %) and children who did not meet recommended intake of protein were 2·26 times more likely to be stunted (AOR = 2·26, 95 % CI: 1·01, 5·05; *P* = 0·047). Cohort study among children less than 5 years old from a rural area in Kenya found that children with a traditional dietary pattern have approximately a 2·5 to 3·1 times higher risk of becoming stunted compared with those with a protein-rich dietary pattern^(37)^.

Regarding weekly food consumption frequency, children who were stunted significantly consumed poultry, eggs and fruits less often than non-stunted children, while consumed legumes more frequently. A previous study investigating association of dietary pattern and stunting reported that dietary intakes of poultry dairy products, dried fruits and nuts were lower among stunted children compared to the non-stunted group^(38)^.

The quantity and nutritional quality of dietary protein well known to affect plasma levels of insulin-like growth factor I, the mediator of growth hormone, also the bone matrix proteins and growth factors, which play important roles in bone formation, are affected by dietary proteins^(39)^.

Dietary habits may have direct consequences on health and diseases through epigenetic processes. Previous study suggested that lower intakes of energy, protein and carbohydrate are significantly associated with increased global DNA methylation in children^(36)^.

The results revealed stunting was associated with sex, mother’s age and education and se level. In relation to mother’s age, the differences in prevalence of stunting decreased with maternal age. The study results corroborate with other studies^(31)^. This may be because younger mothers may tend to have poor knowledge and practices of good nutrition for young children^(31)^.

Regarding SES, children whose families had very low SES were three times more likely to be stunted compared to children whose families had high SES (AOR = 3·05, 95 %CI: 1·45, 6·39; *P* = 0·003). Similarly, previous study showed that wealth status had an inverse relationship with stunting^(31)^.

On the light of this study, it is recommended that multiple measures targeted at reducing child stunting should be taken in a bid to influence policy and conceiving of programmes. Policies and programmes should give greater attention to improving maternal education, especially among younger mothers and improve SES.

Nutrition education messages encouraging high consumption of protein sources including poultry and eggs are recommended. Preventive strategies to prevent stunting and promote adequate and healthy eating are needed.

### Strengths and limitations

Amongst the strengths of this study is the relatively large sample size. This study adds to the literature on stunting and dietary intake in rural settings. Dietary assessment using 24-h dietary recall provides detailed intake data; and relatively small respondent burden (literacy not required). However, there are some limitations. Limitations of current study include using a 24-h dietary recall to collect the dietary data. This method has some advantages in that it is less expensive than dietary records, does not require literacy or a high level of compliance and gives detailed quantitative information about dietary intake. However, it relies on the memory of the informant, depends on an accurate report of the method of preparation, requires accurate estimation of portion sizes and depends on a highly trained and experienced interviewer^(40)^.

Another limitation is that the accuracy of the data depended on the respondent’s memory, honesty and ability to understand the questions.

## Conclusion

Egyptian children who were stunted suffer from poor dietary intake that may play an important role in their linear growth retardation. In this study, we have identified some significant risk factors that predict stunting among Egyptian children. Child-related factors include the child’s sex (being male) and not meeting recommended energetic and protein requirements. Parental/household-related factors include mother’s age and SES. Children with younger mothers and low socio-economic households have been associated with stunting. These results highlight the need for public health intervention programmes that provide access to sufficient, safe and nutritious food and health education focusing on families of low SES. Nutritional education on healthy eating habits and low-cost wholesome food is also recommended.
